# Multimodal neuroimaging alterations in schizophrenia model rats and the modulatory effects of risperidone: a pilot study

**DOI:** 10.3389/fpsyt.2026.1757297

**Published:** 2026-03-27

**Authors:** Shengjie Zhu, Yunxia Liu, Yijie Zhang, Zhiwei Feng, Wenjun Sun

**Affiliations:** 1 The Third Clinical Medical College, Beijing University of Chinese Medicine, Beijing, China; 2 Department of Encephalopathy, Beijing University of Chinese Medicine Third Affiliated Hospital, Beijing, China

**Keywords:** diffusion tensor imaging, hippocampus, magnetic resonance spectroscopy, risperidone, schizophrenia, structural MRI

## Abstract

**Background:**

The hippocampus is considered to be closely associated with the emergence of negative symptoms and cognitive impairments in schizophrenia (SCH). A single imaging modality is insufficient to capture the comprehensive structural, functional, and metabolic alterations in the hippocampus. This study employed multimodal magnetic resonance imaging (MRI) to investigate changes in hippocampal volume, white matter integrity, and metabolite levels in SCH rats, exploring the positive regulatory effects of risperidone.

**Methods:**

Ten pregnant Wistar rats were randomly divided into two groups on gestational day 9: one was injected with poly(I:C) to establish SCH model, and the other received saline. Nine male offspring were selected and randomly assigned to three groups (n=3):Normal(from saline grop), SCH, and SCH+Risperidone (Treated) group (from poly(I:C)). From 76 days of age, the Treated group was administered risperidone solution by gastric lavage, whereas the other two groups received distilled water, for a 28-day period. Thereafter, multimodal MRI scans were conducted: Structural MRI (sMRI) for bilateral hippocampal volume; diffusion tensor imaging (DTI) for hippocampal FA and ADC values; and magnetic resonance spectroscopy (MRS) for metabolite-to-creatine ratios: N-acetylaspartate to creatine (NAA/Cr), choline to creatine (Cho/Cr), glutamate to creatine (Glu/Cr), myo-inositol to creatine (mI/Cr).

**Results:**

Hippocampus Volume: Compared to the normal group, the SCH group exhibited a significant reduction in bilateral hippocampus volume (*P* < 0.05). Risperidone-treatment significantly increased bilateral volume compared to the SCH group (*P* < 0.05). DTI: In the SCH group, FA was decreased in the left (*P* < 0.05) and right (*P* < 0.01) hippocampus compared to normals. Conversely, ADC was increased in the right hippocampus (*P* < 0.05). Following risperidone treatment, the FA was significantly increased in both hippocampus (*P* < 0.05). MRS: The SCH group showed lower ratios of NAA/Cr and Glu/Cr than the normal group (*P* < 0.05). Following risperidone treatment, the NAA/Cr was significantly elevated (*P* < 0.01).

**Conclusions:**

The Poly(I:C)-induced schizophrenia rat model exhibits reduced hippocampal volume, neuronal damage, impaired white matter integrity, and glutamatergic system dysfunction. These preliminary findings suggest a potential modulatory effect of risperidone, warranting validation in larger cohorts.

## 1 Introduction

Schizophrenia (SCH) is a severe and prevalent mental disorder whose exact pathophysiological mechanisms remain unclear. It typically emerges in early adulthood and is characterized by disruptions in perception, thought, emotion, and behavior. According to the World Health Organization, the global lifetime prevalence of schizophrenia is approximately 0.32% (1). The disorder is considered one of the most severe mental illnesses. Its core symptoms are categorized into three groups: positive symptoms (such as hallucinations, delusions, and agitation), negative symptoms (including apathy, social withdrawal, and reduced motivation), and cognitive impairment. This cognitive decline primarily involves deficits in executive function, memory, and attention, and can include varying degrees of intellectual disability. Notably, cognitive impairment is particularly challenging to manage in clinical practice and has emerged as a leading cause of functional disability in patients with schizophrenia (2).

Advances in neuroimaging have been instrumental in visualizing the pathophysiology of schizophrenia and the pharmacological actions of antipsychotic drugs. Early structural MRI studies revealed common anatomical alterations in schizophrenia, including volume reductions in the prefrontal and temporal cortices and hippocampus, alongside enlarged lateral ventricles (3). However, significant heterogeneity across studies has limited the diagnostic utility of conventional structural imaging. Multimodal MRI techniques offer a more sensitive and specific means of identifying white matter (WM) damage, disrupted brain network connectivity, and aberrant cerebral metabolism in schizophrenia, making them a powerful tool for investigating its pathogenesis, aiding diagnosis, and evaluating treatment response (4). Diffusion Tensor Imaging (DTI) could be used to assess the integrity and connectivity of white matter tracts in various brain regions of individuals with schizophrenia. Studies have identified reduced fractional anisotropy (FA) in patients within regions such as the corona radiata, internal capsule, and frontal-parietal lobes (5), indicating impaired white matter integrity and disrupted neural connectivity. This white matter damage in critical areas may explain the emergence of cognitive deficits in schizophrenia (6). Jiang et al. identified disruptions in specific WM network nodes, including the corpus callosum, optic radiation, and posterior corona radiata and tempo-occipital WM tracts, in SCH. Furthermore, they reported the clustering coefficient, local efficiency and synchronization of the WM network were negatively correlated with negative symptoms (7). Magnetic resonance spectroscopy (MRS) is a non-invasive imaging technique that measures the levels of various neuro-metabolites in brain tissue, allowing for a biochemical analysis of neurons in SCH. Studies have shown that levels of N-acetylaspartate (NAA) are reduced in the frontal lobe and hippocampus of individuals with schizophrenia (8). Furthermore, Allen et al. found that individuals at clinical high risk for SCH exhibit lower glutamate(Glu) levels in the thalamus compared to healthy controls, which may be associated with poorer clinical outcomes (9).

Risperidone, a second-generation antipsychotic, could ameliorate positive symptoms, negative symptoms, and cognitive impairment in SCH (10). However, some patients with schizophrenia do not respond fully to antipsychotic medications. Previous research indicates that short-term risperidone treatment is associated with increased gray matter volume in several regions, including the basal ganglia, inferior parietal lobule, and right prefrontal, temporal, and occipital lobes, with the most pronounced changes observed in the basal ganglia (11). In a study of 42 first-episode schizophrenia patients, DTI and functional MRI (fMRI) were used to map the default mode network (DMN), revealing impaired functional connectivity between the posterior cingulate cortex/precuneus (PCC/PCUN) and the medial prefrontal cortex (mPFC). Following 8 weeks of risperidone monotherapy, the functional connectivity between these regions increased, a change that correlated with an improvement in positive symptoms (12). Animal studies have shown that offspring of maternal rats exposed to Poly(I:C) during pregnancy develop structural abnormalities relevant to schizophrenia, such as enlarged lateral ventricles and a smaller hippocampus. When treated with risperidone throughout adolescence, these abnormalities were absent in adulthood (13). Furthermore, risperidone treatment has been linked to improved cerebral metabolism in schizophrenia, evidenced by elevated glutamate levels in the anterior cingulate cortex (ACC) (14). Based on prior research, Poly(I:C)-induced SCH model rats may exhibit alterations at the structural, microstructural, and metabolic levels in the hippocampus, and chronic treatment with risperidone improves these deficits. Therefore, the primary objective of this study is to comprehensively characterize these neuroimaging alterations in the hippocampal region of SCH model rats using a multimodal MRI approach—including structural MRI (sMRI), diffusion tensor imaging (DTI), and magnetic resonance spectroscopy (MRS). A secondary objective is to observe the effects of 28 days of risperidone treatment on hippocampal morphology, white matter integrity, and metabolite levels, and explore the drug’s regulatory role in brain structural and metabolic abnormalities in a rat model of schizophrenia. Given the complexity and high cost of multimodal imaging, this study serves as a pilot experiment to preliminarily explore the brain imaging patterns in Poly(I:C)-induced rat models and the potential effects of drug intervention, thereby providing foundational data for subsequent large-scale hypothesis-testing research.

## 2 Materials

### 2.1 Animals

All experimental procedures in this study were conducted in accordance with the ARRIVE guidelines and were approved by the Animal Ethics Committee of Beijing University of Chinese Medicine (Approval No. BUCM-2023071405-3235). Ten pregnant female Wistar rats were obtained on gestational day 6 from Beijing Speifu Biotechnology Company, Ltd. These dams were of the same origin, age, and weight. All animals were housed in a standard specific pathogen-free (SPF) environment at Beijing University of Chinese Medicine, maintained at 20–22 °C with a 12-hour light/dark cycle (lights on from 8:00 AM to 8:00 PM). Food and water were available ad libitum.

### 2.2 Reagents and drugs

Polyinosinic-polycytidylic acid [Poly(I:C)] was purchased from Sigma-Aldrich. Immediately before use, it was dissolved in 0.9% sterile saline to prepare the injection solution. Risperidone oral solution (30 mL: 30 mg; National Drug Approval Number: J20180022) was obtained from Xi’an Janssen Pharmaceutical Ltd. For administration, the solution was diluted in distilled water to create a risperidone mixture.

### 2.3 Major instruments and equipment

*In vivo* magnetic resonance imaging was performed using a 7.0T scanner (Bruker, Germany) located in the Small Animal Imaging Laboratory of the Experimental Center at Capital Medical University.

## 3 Methods

### 3.1 Model establishment

The maternal immune activation (MIA) method was employed to generate a schizophrenia model, with timing set at gestation day 9 (GD9) in the dam. This critical period of embryonic development is characterized by the activation of inflammatory responses, which can cause irreversible damage to the nervous system of offspring rats (15). The dams were randomly assigned to one of two groups: the MIA group received a single intravenous tail vein injection of Poly(I:C) (10 mg/kg), dissolved in 0.9% sterile saline. The normal group received an equivalent volume of saline only. The day of delivery is designated as postpartum day 0 (PD0). The offspring were weaned at PD21 and reared separately by sex, with male offspring selected for subsequent experiments. A series of behavioral tests were conducted on the offspring from days 61 to 74 post-delivery, confirming successful modeling (16).

### 3.2 Animal grouping and drug administration

Three offspring from saline-injected dams were randomly assigned to the normal group. Following the confirmation of successful schizophrenia model induction via behavioral tests, three offspring from Poly(I:C)-injected dams were randomly assigned to the schizophrenia model (SCH) group, and another three to the risperidone treatment (Treated) group. Group allocation was performed by an investigator not involved in behavioral testing or subsequent data analysis. Oral gavage began on postnatal day 75. The Treated group received risperidone mixture at a concentration of 0.04 mg/mL and a dosage of 0.4 mg/kg/day. The normal and SCH groups received an equivalent volume of distilled water. Adjust the gavage dose based on weekly body weight changes in rats, followed by magnetic resonance imaging after 4 weeks of gavage.

### 3.3 Measurement protocols

#### 3.3.1 Structural MRI

Anesthetized rats were secured on the instrument platform and scanned using a 7.0T MRI spin-echo sequence to acquire T2-weighted images (T2WI). Scan parameters were as follows: Repetition time (TR) = 4200 ms, Echo time (TE) = 36 ms, Matrix (MTX) = 256×256, Slice thickness (ST) = 0.8 mm, Inter-slice gap = 1 mm, 30 slices total, Field of view (FOV) = 3.5 cm × 3.5 cm. The acquired images were imported into ITK-SNAP software. The hippocampal shape was then manually contoured layer by layer to obtain hippocampal volume data.

#### 3.3.2 Diffusion tensor imaging

Rats were scanned in a coronal orientation using a DTI sequence. The parameters were: TR = 6752 ms, TE = 22 ms, FOV = 3.5 × 3.5 cm, matrix = 128 × 128, number of excitations (NEX) = 1, slice thickness = 1.00 mm, with 30 diffusion gradient directions using b-values of 0 and 1000 s/mm². Acquired using a single-excitation spin-echo planar sequence to measure fractional anisotropy (FA) and apparent diffusion coefficient (ADC).

#### 3.3.3 Magnetic resonance spectroscopy

In the bilateral hippocampal regions, select a 3 mm × 3 mm × 3 mm cubic region of interest (ROI) in each. Following field-equalization and water suppression, acquire the 1H spectrum using the PRESS sequence with the following parameters: spectral width = 1500 Hz, data points = 2048, TR = 2500 ms, TE = 20 ms, 500 repetitions. The acquired 1H spectrum underwent Fourier transform and baseline correction, with the chemical shift of the water peak designated as 4.77 ppm. This allowed determination of the following chemical shifts: NAA at 2.02 ppm, Cr at 3.0 ppm, Cho at 3.22 ppm, Glu at 2.35 ppm, and mI at 3.56 ppm. The data were then imported into Paravision and analyzed using configured TopSpin 2.0 software (Bruker BioSpin, USA) to obtain metabolite spectra. After baseline correction, the areas under the metabolite curves corresponding to the water peak were calculated to determine the NAA/Cr, Cho/Cr, Glu/Cr, and mI/Cr ratios, followed by statistical analysis.

### 3.4 Statistical analysis

All statistical analyses were performed using SPSS software (version 26.0). Continuous variables that were normally distributed are described as the mean ± standard deviation (mean ± SD). Comparisons across multiple groups were conducted using one-way analysis of variance (ANOVA). If the assumption of homogeneity of variance was met, *post-hoc* pairwise comparisons were performed using the LSD test; otherwise, the Tamhane’s T2 test was applied. For data that violated the assumption of normal distribution, the non-parametric independent-samples Kruskal-Wallis test was used. *P*-values < 0.05 were considered statistically significant. To complement the null hypothesis significance testing, we calculated effect sizes (partial eta-squared) for the main comparisons to estimate the magnitude of the observed differences. The results are interpreted primarily as descriptive trends that require confirmation in future studies with larger sample sizes.

## 4 Results

### 4.1 Hippocampal volume changes

**Figure 1:**
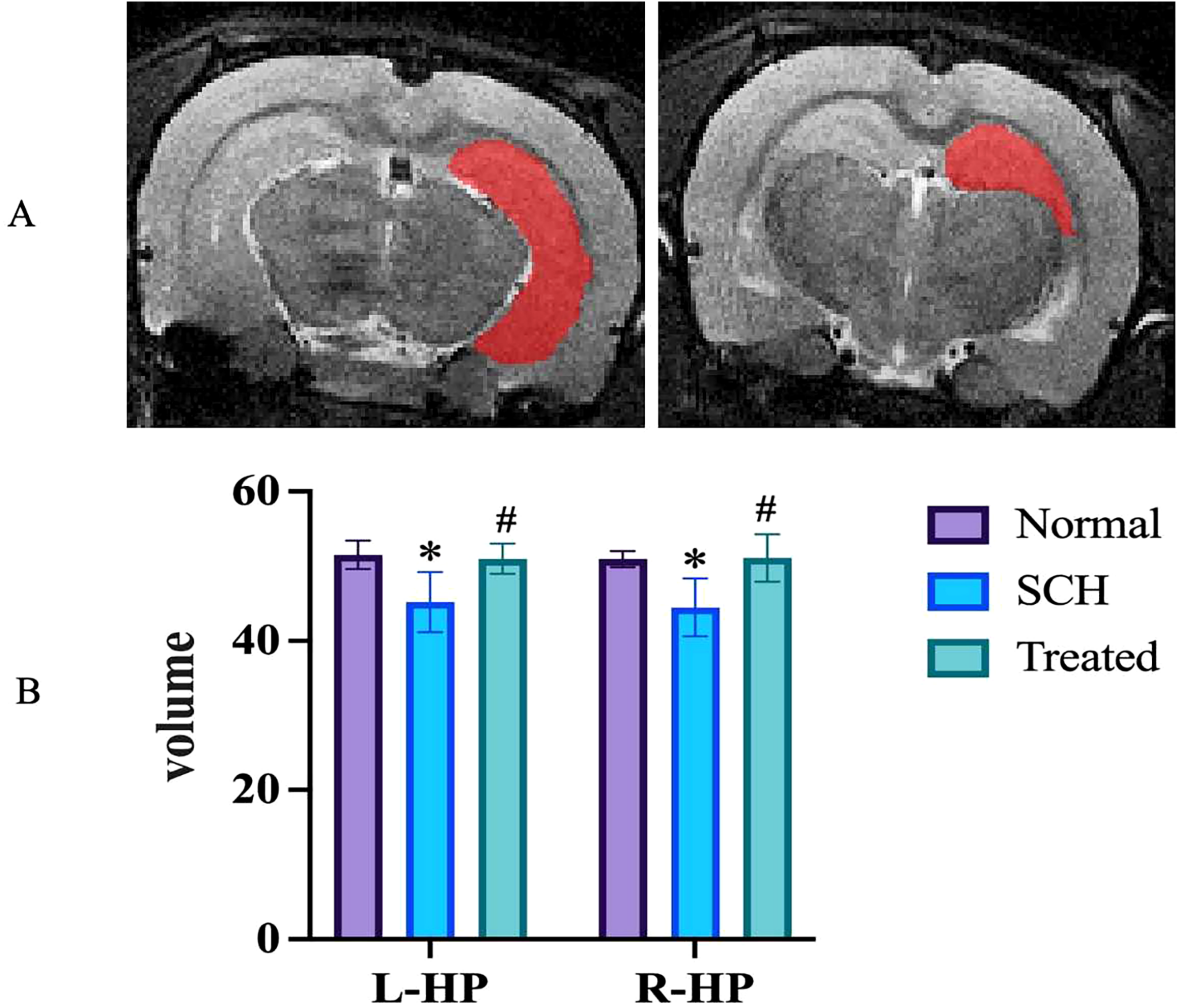
Hippocampal volume. **(A)** Hippocampal ROI acquisition via sMRI technology; **(B)** Bilateral hippocampal (HP) volume comparison. L = left; R = right. Data are expressed as mean ± SD. *P < 0.05 vs. Normal; #*P* < 0.05 vs. SCH. (n = 3 in Normal group, n = 3 in SCH group, n = 3 in Treated group. Treated denotes the risperidone treatment group; SCH denotes the schizophrenia rat model group).

The volume of the bilateral hippocampus was quantified from T2-weighted images (T2WI) acquired via sMRI, using ITK-SNAP software for segmentation (**Figures 1A, B**). Compared to the normal group, the schizophrenia model (SCH) group exhibited a significant reduction in the volume of both the left and right hippocampus (*P* < 0.05). Following risperidone treatment, hippocampal volume was significantly increased bilaterally compared to the SCH group (*P* < 0.05). **Table 1** presents the imaging metrics of the hippocampi from three groups of rats.

**Table 1:** Comparison of hippocampal imaging characteristics among three groups of rats.

Measures	Group	Means	Standard deviations	P	η2
Left volume	Normal	51.54	1.9		0.607
	SCH	45.22	4.02	*0.033	
	Treated	51	2	#0.046	
Right volume	Normal	50.95	1.09		0.616
	SCH	44.5	3.88	*0.041	
	Treated	51.43	3.44	#0.032	
Left FA	Normal	0.149	0.019		0.676
	SCH	0.113	0.006	*0.017	
	Treated	0.143	0.011	#0.032	
Right FA	Normal	0.147	0.003		0.749
	SCH	0.128	0.009	**0.008	
	Treated	0.144	0.004	#0.014	
Left ADC	Normal	0.00075433	0.000009018		0.601
	SCH	0.00079733	0.000033471		
	Treated	0.00075367	0.000006658		
Right ADC	Normal	0.00074467	0.000008737		0.611
	SCH	0.000791	0.000029309	*0.022	
	Treated	0.00076733	0.000009452		
NAA/Cr	Normal	1.198533	0.0762642		0.75
	SCH	0.9703	0.0435248	*0.014	
	Treated	1.229733	0.1111827	##0.008	
Glu/Cr	Normal	0.421267	0.064129		0.514
	SCH	0.288633	0.0557769	*0.048	
	Treated	0.377133	0.0756353		
CHO/Cr	Normal	0.955767	0.1522386		0.279
	SCH	0.9917	0.2273936		
	Treated	0.7006	0.347235		
mI/Cr	Normal	0.094267	0.0472149		
	SCH	0.125967	0.0487853		
	Treated	0.04535	0.0393858		

*P < 0.05, **P < 0.01 vs. Normal; #*P* < 0.05, ##*P* < 0.01 vs. SCH.

### 4.2 Bilateral hippocampal DTI

**Figure 2:**
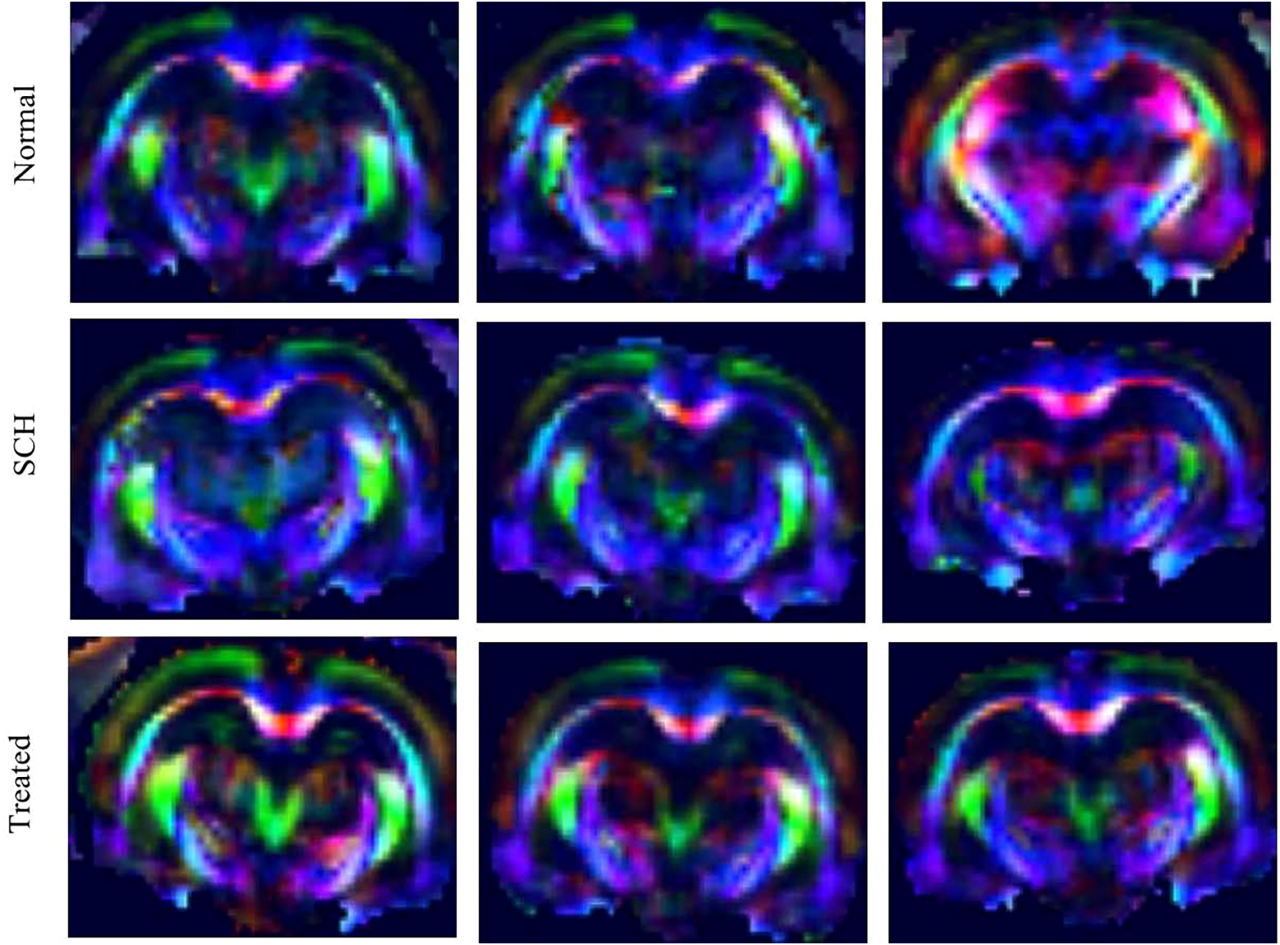
DTI scans. Representative color-coded fractional anisotropy (FA) maps from the Normal, SCH, and Treated groups, acquired via DTI.

**Figure 3:**
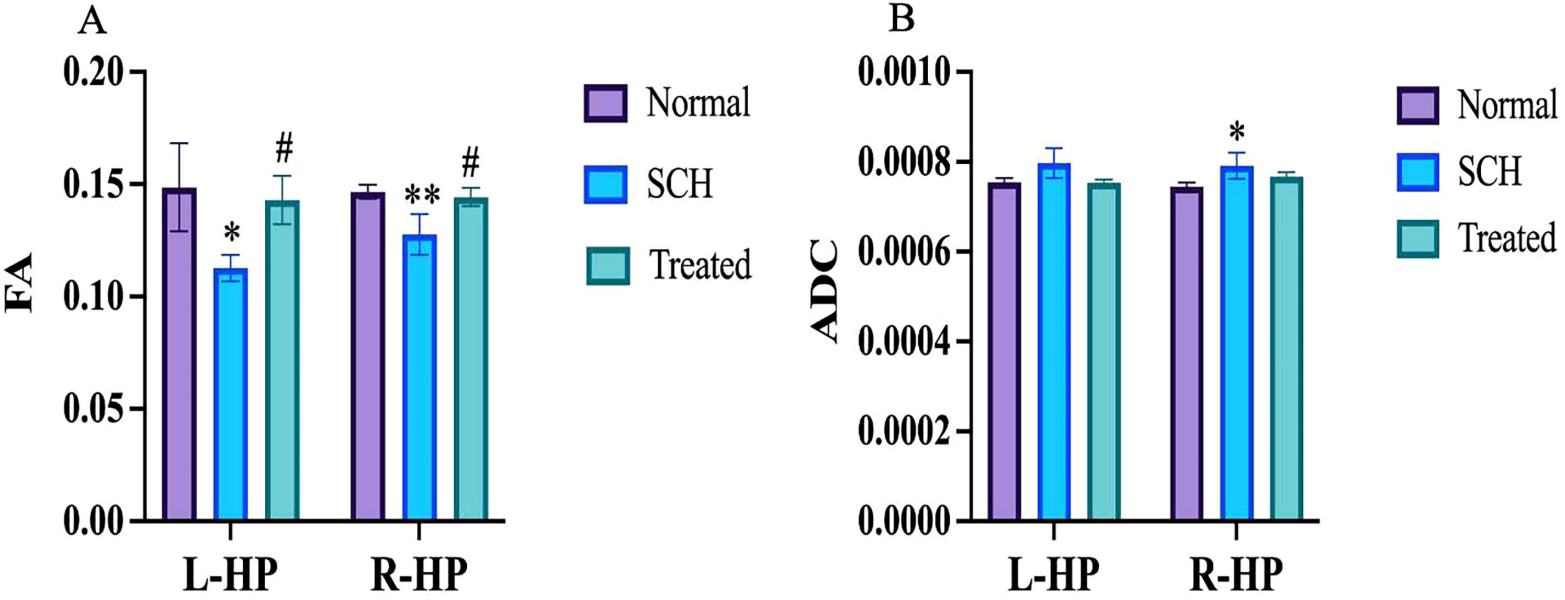
Changes in fractional anisotropy (FA) and apparent diffusion coefficient (ADC) values. **(A)** FA values in the bilateral hippocampus (HP). **(B)** ADC values in the bilateral HP. Data are presented as mean ± SD. *P < 0.05 and **P < 0.01 versus the Normal group; #*P* < 0.05 versus the SCH group.

DTI was used to assess white matter integrity in the bilateral hippocampus by measuring fractional anisotropy (FA) and apparent diffusion coefficient (ADC) within defined regions of interest (ROIs). **Figure 2** shows color-coded fiber tractography maps for the Normal, SCH, and Treated groups. As shown in **Figure 3A**, the SCH group exhibited significantly lower FA values in the hippocampus compared to the Normal group (left: *P* < 0.05; right: *P* < 0.01). Following risperidone treatment, FA values were restored to near-normal levels, showing no significant difference from the Normal group (*P* > 0.05) but a significant increase compared to the SCH group (*P* < 0.05). Regarding ADC values (**Figure 3B**), the SCH group showed a significant increase in the right hippocampus compared to the Normal group (*P* < 0.05), with a non-significant upward trend in the left hippocampus. The Treated group exhibited ADC values comparable to the Normal group. Although ADC values in the Treated group were slightly lower than in the SCH group, this difference was not statistically significant.

### 4.3 Bilateral hippocampal MRS

**Figure 4:**
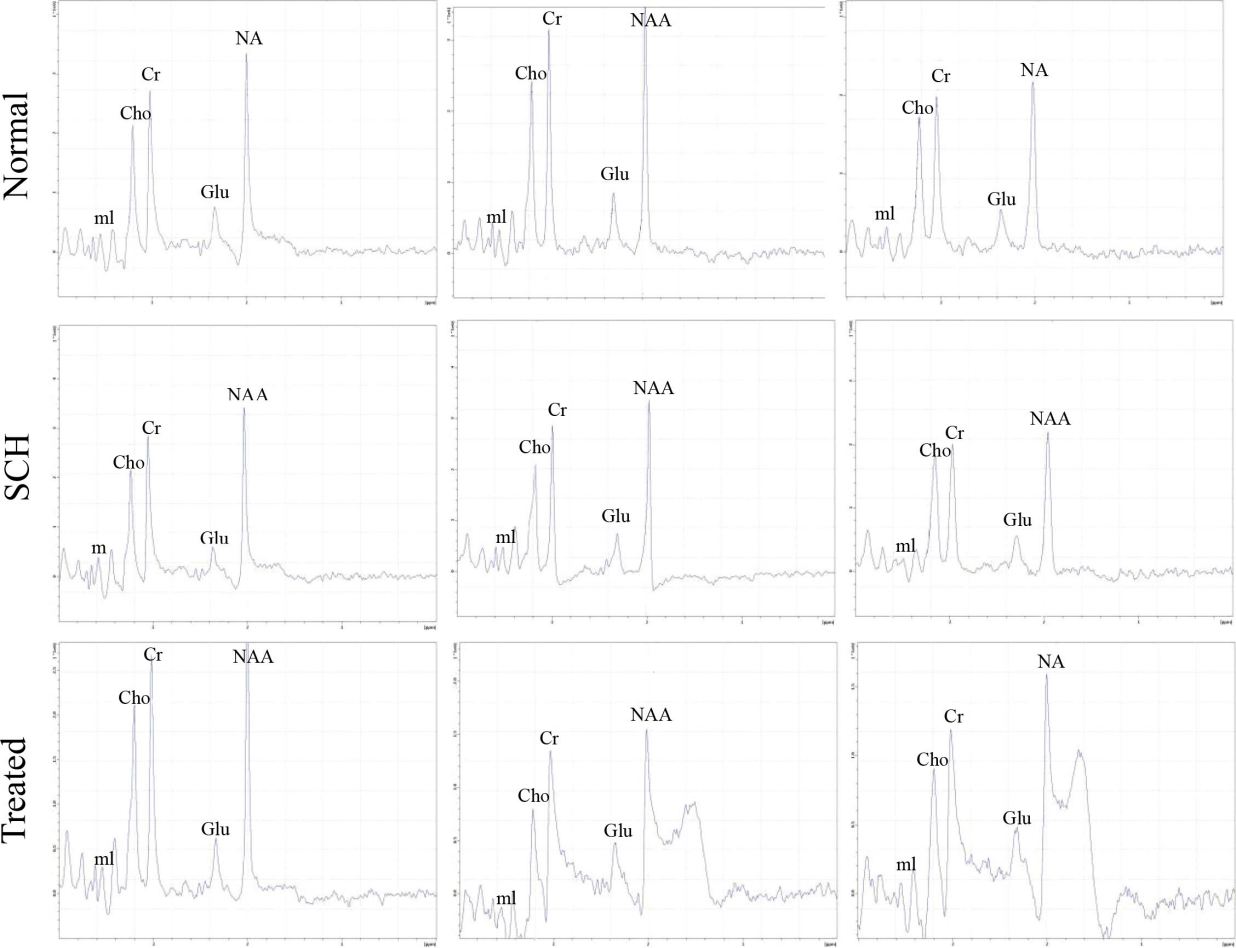
MRS of HP of rats in normal group, SCH group and treated group.

**Figure 5:**
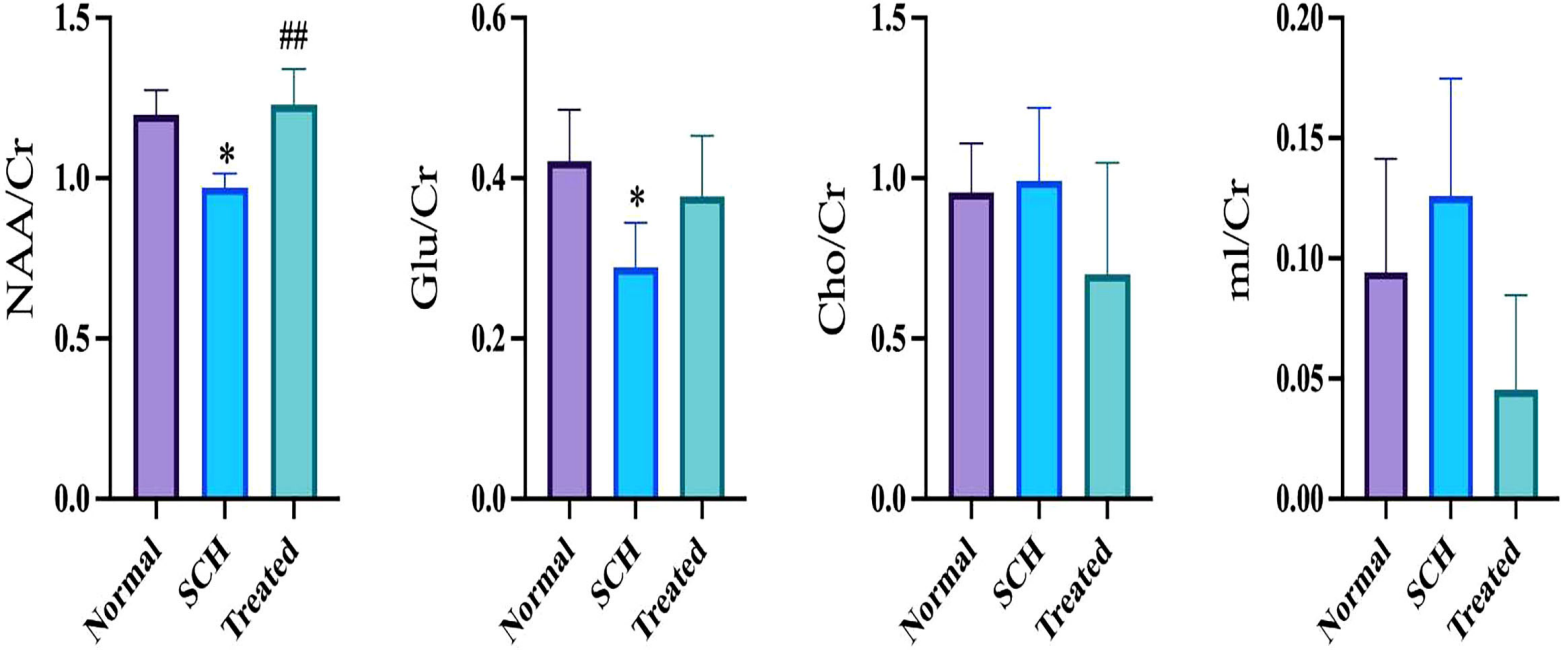
Comparison of metabolite levels in hippocampal brain tissue, including N-acetylaspartate to creatine (NAA/Cr), choline to Cr (CHO/Cr), glutamate to Cr (Glu/Cr) and inositol to Cr (mI/Cr). Data are expressed as mean ± SD. *P < 0.05 vs. Normal; #*P* < 0.05, ##*P* < 0.01 vs. SCH.

Metabolite levels in the bilateral hippocampus were quantitatively analyzed across the three groups using magnetic resonance spectroscopy (MRS) (**Figure 4**). Compared to the Normal group, the SCH group exhibited significantly lower hippocampal ratios of N-acetylaspartate to creatine (NAA/Cr) and glutamate to creatine(Glu/Cr) (*P* < 0.05). Risperidone treatment significantly increased the NAA/Cr ratio compared to the SCH group (*P* < 0.01). Although the Glu/Cr ratio also increased in the treatment group, this change was not statistically significant (**Figure 5**).

## 5 Discussion

This study aimed to explore neuroimaging alterations in the hippocampal region of a Poly(I:C)-induced SCH rat model and to elucidate the potential modulatory effects of risperidone. Our findings reveal significant hippocampal pathology in SCH rats, including volume reduction, white matter fiber bundle damage, and cerebral metabolic disturbances. Risperidone partially ameliorated hippocampal damage in SCH rats by restoring hippocampal volume, repairing white matter fiber integrity, and improving metabolite levels in hippocampal tissue.

Schizophrenia can result in cognitive and emotional impairments, including memory loss, diminished executive function, and emotional blunting. The hippocampus, primarily involved in memory and emotional regulation (17), is considered crucial in the pathology of schizophrenia. Consequently, the patients with schizophrenia frequently demonstrate alterations in hippocampal morphology and function (18, 19). Poly(I:C) is a double-stranded RNA (dsRNA) synthesis analog composed of homopolymeric chains of polyinosinic acid and polycytidylic acid. It induces the expression of proinflammatory factors, stimulating cellular immune responses involving neurons and glial cells via the circulatory system and blood-brain barrier. The hippocampus contains abundant microglia, whose dysfunction may lead to neuronal injury (20). Research indicates that the early gestation period (days 8.5 to 9.5) in rats represents a critical window for fetal neural development. Poly(I:C)-induced maternal immune responses cause inflammatory damage to fetal neurons and glial cells (particularly microglia), impairing hippocampal development and triggering a spectrum of psychiatric disorders (21). Treatment with the atypical antipsychotic risperidone can improve abnormalities in hippocampal neuronal development, abnormal microtubule protein expression, and disruption of the vascular network in offspring resulting from maternal immune activation in pregnant rats (22). Therefore, this study employed a maternal immune activation approach to induce a schizophrenia rat model using Poly(I:C). Multimodal neuroimaging evaluations—including sMRI, DTI, and MRS—were conducted around the hippocampus to analyze the potential effects by which risperidone reduces neuroinflammation and improves hippocampal damage.

### 5.1 Hippocampal volume

Reduced hippocampal volume is a consistent finding in neuroimaging studies of schizophrenia, reported across multiple investigations (19, 23, 24). Our study found that Poly(I:C)-induced SCH rats exhibited significant bilateral hippocampal volume reduction, consistent with most previous findings (25, 26). This may be associated with increased expression of proinflammatory molecules within hippocampal cells and neuronal damage in adult SCH rats (27). Additionally, our group’s prior research revealed that offspring born to Poly(I:C)-exposed dams exhibit severe cognitive impairments. Cognitive deficits have long been recognized as a consistent and stable feature of schizophrenia, likely closely associated with hippocampal atrophy and overall volume reduction. Risperidone is an anti-inflammatory drug with clear therapeutic effects on both positive and negative symptoms in schizophrenia patients. It demonstrates good therapeutic efficacy in Poly(I:C)-induced schizophrenia rat models. We found that following risperidone administration, bilateral hippocampal volumes in SCH rats increased significantly. Studies suggest that low-to-moderate doses of risperidone treatment do indeed improve hippocampal volume (28). Sasa et al. similarly reported significant increases in left hippocampal and bilateral thalamic volumes in schizophrenia patients after four weeks of risperidone treatment (29). However, some reports indicate reduced hippocampal volume in normal groups and MIA offspring treated with antipsychotics like risperidone, potentially related to the high risperidone dose (1.2 mg/kg) used in these studies (27, 30).

### 5.2 Hippocampal DTI

DTI is an unique non-invasive method for detecting the integrity and connectivity of white matter tracts at the molecular level. It can reveal crucial information linking white matter tracts, disconnection mechanisms, and clinically relevant symptoms. White matter damage may impair cognitive processes in schizophrenia (31). The FA reflects the anisotropy of water diffusion within white matter fibers, indirectly indicating the integrity of axons and myelin sheaths. It is highly sensitive to the microstructural integrity of white matter tracts (32). Reduced FA values indicate neuronal degeneration, fiber/axonal disruption, or demyelination changes (33). ADC represents the speed and extent of water molecule diffusion within the observed region. Under normal conditions, ADC magnitude primarily correlates with cellular density. As two distinct parameters reflecting diffusion motion, lower restrictions on water molecule diffusion within tissue result in higher ADC values and lower FA values (34). Therefore, within the nervous system, elevated ADC values may indicate reduced neuronal density, suggesting neuronal damage or apoptosis.

We found that compared to the normal group, SCH rats exhibited significantly lower FA values in the bilateral hippocampal regions, a finding consistent with other reports (35, 36). This indicates substantial white matter damage in the hippocampus, involving impaired integrity and disconnection of neural fibers. One of the most reliable observations in this context is reduced FA alongside an increased ADC in the corpus callosum (37). Our findings show consistency between reduced FA values and increased ADC values in the right hippocampus, whereas the left hippocampus exhibits decreased FA without significant ADC changes. This discrepancy may relate to glial cell proliferation and migration/infiltration into enlarged extracellular spaces in this region (29), where inflammatory mechanisms likely play a more prominent role, potentially activating excessive microglial proliferation (38). In the Treated group, FA values were significantly higher in the bilateral hippocampi compared to the SCH group, while ADC values showed no significant difference. This indicates that SCH rats exhibit structural abnormalities in neurons and fiber tracts within both hippocampi, and risperidone may repair hippocampal microstructural damage by reducing inflammatory or immune responses (39).

### 5.3 Hippocampal MRS

This study employed MRS technology to measure metabolite concentrations in the hippocampal region of SCH rats and examined the intervention effects of risperidone on the onset and progression of schizophrenia from a neurometabolic perspective.

NAA serves as a marker of neuronal and axonal structural integrity, reflecting neuronal cell death or dysfunction. This study observed a significant decrease in the NAA/Cr ratio in the bilateral hippocampal regions of SCH model rats, potentially indicating reduced neuronal density, loss of integrity, and impaired spatial learning and memory abilities in SCH rats (6, 40). Glutamatergic dysfunction is closely associated with schizophrenia. We observed a significant decrease in the Glu/Cr ratio in the hippocampus of the SCH group. which may be partially attributable to suppressed N-methyl-D-aspartate (NMDA) receptor function leading to diminished glutamate activity or disrupted glutamate-glutamine homeostasis (involved in neurotransmitter inactivation and regulatory processes) (41, 42). Glutamate (Glu) is an excitatory neurotransmitter, and its decreased concentration may be associated with the more prominent negative symptoms observed in schizophrenia. This finding is consistent with our previous study, which documented reduced locomotor activity, blunted responsiveness, and cognitive decline in male SCH rats (16). Cho, a precursor to the neurotransmitter acetylcholine, forms the pathophysiological basis for affective disorders and participates in phospholipid metabolism within neuronal membranes. Its elevation may be associated with impaired cellular transport and damage to lipid structures in cell membranes, myelin sheaths, and the brain (43). mI is considered a marker of glial cells, and its elevation typically reflects neuroinflammation, neuronal injury, and glial cell proliferation (44). Our study found that compared to the normal group, Cho/Cr and mI/Cr were elevated to varying degrees in schizophrenia rats, though the differences were not significant. Similar findings have been reported in other studies (45). Following risperidone treatment in the SCH rats, we observed a significant increase in the NAA/Cr ratio. The Glu/Cr ratio also showed an increasing trend, while the Cho/Cr and mI/Cr ratios displayed decreasing trends; however, these latter changes were not statistically significant. Collectively, these findings suggest that risperidone may facilitate the repair of neurons damaged by inflammatory stimuli. This restorative effect could be achieved through promoting neural remodeling, restoring neuronal integrity, modulating the glutamatergic system, and normalizing metabolic activity in cell membranes.

Although this study provides initial insights into the multimodal imaging changes in the hippocampus of a schizophrenia rat model and the interventive effects of risperidone, several limitations should be considered. The primary limitation stems from the exploratory nature of this multimodal imaging study and the practical constraints of animal experimentation, which restricted sample size to three per group. This small sample size inherently limits the statistical power and generalizability of the findings. This lowers statistical power, potentially failing to detect genuinely existing subtle differences (increasing the risk of Type II errors), and makes results susceptible to outliers, limiting the extrapolation of conclusions. Nevertheless, as an exploratory effort, this study provides valuable reference data for future research by calculating effect sizes, aiding subsequent studies in conducting precise sample size estimations. Therefore, the conclusions presented herein should be regarded as preliminary, hypothesis-generating findings awaiting validation. Second, the Poly(I:C)-induced SCH rat model, while effectively simulating certain negative symptoms and cognitive deficits, cannot fully replicate the entire spectrum of human schizophrenia, which is a highly heterogeneous and complex disorder. Third, different hippocampal subregions may exhibit distinct pathological alterations and drug responses. However, constrained by resolution limitations and post-processing methods, this study could not perform precise subregional analysis, presenting only overall average effects across the hippocampus. Fourth, the study administered risperidone at a single time point and dose. Further exploration is needed to determine whether different doses or administration schedules of the intervention would induce distinct imaging changes. To address the aforementioned limitations, the research team will continue to expand the sample size, refine technical shortcomings in image analysis, and further investigate the imaging changes and brain network connectivity across different brain regions in SCH rats. Additionally, the experimental design will be enhanced by establishing multiple risperidone dosage groups and varying administration durations to comprehensively evaluate the time-dose relationship of risperidone. This will provide more precise imaging references for clinical treatment protocols.

While the present study was conducted in a rat model of schizophrenia, our findings hold potential translational value for human clinical practice. Multimodal neuroimaging biomarkers have been consistently implicated in schizophrenia pathophysiology and treatment response in human studies. By demonstrating that risperidone partially reverses these abnormalities in a well-validated maternal immune activation model, our results support the use of such imaging metrics as sensitive indicators of neurobiological treatment effects. Importantly, Future studies employing graded doses of risperidone and variable treatment durations in this model could help identify the minimum effective dose or optimal treatment window for reversing hippocampal pathology. Such data could eventually inform personalized dosing strategies in schizophrenia patients.

## 6 Conclusion

This study employed a multimodal imaging approach—combining sMRI, DTI, and MRS—to investigate neuropathological changes in the hippocampus of a Poly(I:C)-induced SCH rat model. Furthermore, it characterized the imaging signatures associated with the neuroprotective effects of risperidone. Our results revealed that Poly(I:C) -induced SCH rat led to hippocampal atrophy, neuronal injury, white matter impairment, and glutamatergic system dysfunction. Risperidone exhibits potential regulatory effects on brain structural and metabolic abnormalities in the SCH rat model. These findings provide macro-imaging insights into understanding the clinical efficacy of risperidone, while the underlying molecular mechanisms warrant further investigation.

## Data Availability

The original contributions presented in the study are included in the article/supplementary material. Further inquiries can be directed to the corresponding author.
